# The Metabolomic Profile of Spent Culture Media from Day-3 Human Embryos Cultured under Low Oxygen Tension

**DOI:** 10.1371/journal.pone.0142724

**Published:** 2015-11-12

**Authors:** Maria José de los Santos, Pilar Gámiz, José María de los Santos, Josep Lluís Romero, Nicolás Prados, Cristina Alonso, José Remohí, Francisco Dominguez

**Affiliations:** 1 IVI Valencia, Valencia, Spain; 2 INCLIVA Biomedical Research and Fundación IVI, Valencia, Spain; 3 IVI Sevilla, Sevilla, Spain; 4 OWL, Derio, Spain; Peiking university third hospital, CHINA

## Abstract

Despite efforts made to improve the in vitro embryo culture conditions used during assisted reproduction procedures, human embryos must adapt to different in vitro oxygen concentrations and the new metabolic milieu provided by the diverse culture media used for such protocols. It has been shown that the embryo culture environment can affect not only cellular metabolism, but also gene expression in different species of mammalian embryos. Therefore we wanted to compare the metabolic footprint left by human cleavage-stage embryos under two types of oxygen atmospheric culture conditions (6% and 20% O_2_). The spent culture media from 39 transferred and implanted embryos from a total of 22 patients undergoing egg donation treatment was analyzed; 23 embryos came from 13 patients in the 6% oxygen concentration group, and 16 embryos from 9 patients were used in the 20% oxygen concentration group. The multivariate statistics we used in our analysis showed that human cleavage-stage embryos grown under both types of oxygen concentration left a similar metabolic fingerprint. We failed to observe any change in the net depletion or release of relevant analytes, such as glucose and especially fatty acids, by human cleavage-stage embryos under either type of culture condition. Therefore it seems that low oxygen tension during embryo culture does not alter the global metabolism of human cleavage-stage embryos.

## Introduction

Since the very start of the use of in vitro fertilization (IVF) protocols, embryos and gametes have been cultured at atmospheric oxygen levels as standard procedure in most laboratories. Data showing that the oxygen partial pressure conditions in the human genital reproductive tract are very low [1–4] therefore suggest that this laboratory condition is suboptimal, and that culturing embryos at a low oxygen concentration may be an important way to improve clinical outcomes following IVF cycles. Indeed, a recent meta-analysis of the effects of low oxygen tension during embryo culture concluded that these conditions improve pregnancy and live birth rates in patients using their own oocytes [5].

The embryonic culture environment can affect not only cellular metabolism but also gene expression in different species of mammalian embryos [6–9]. For instance, low oxygen culture systems alter glycolytic metabolism in pig blastocysts, shifting the tricarboxylic acid pathway to the anabolic pentose phosphate pathway and to lactic acid production [7], thus decreasing the amino acid uptake rate [4]. In mouse embryos it downregulates a group of ten proteins/biomarkers each with masses between 4 and 20 kDa [10].

Regarding gene expression patterns, low oxygen culture conditions decrease the expression of mRNA stress indicator genes such as heat shock proteins [11, 12] or manganese superoxide dismutase (MnSOD) [13] in both cows and mice. Furthermore, hypoxia is able to alter the methylation status of a variety of cells including skin fibroblasts, tumor cells, and trophoblast cells [14–16]. However, no data is yet available concerning the effect of a low oxygen atmosphere on the global metabolic profile of human embryos exposed to these culture conditions.

The metabolomics field has recently developed new technologies and methodologies which can perform global quantitative and qualitative analysis of the metabolites produced and secreted by any cell, tissue, or even simple organisms [17, 18]. Low-molecular-weight metabolites are the final products of cellular metabolism and therefore could shed some light on the changes produced in a closed system when a variety of genetic, nutritional, and environmental conditions occur [17]. These non-invasive quantitative techniques are used to study the metabolic status of the embryo by using embryo-spent culture media to predict embryo viability and the likelihood of pregnancy. In recent years research has increasingly focused on comparing the metabolic fingerprint of fresh and vitrified embryos [19]. Spectroscopic techniques such as Mass Spectrometry (MS) and Nuclear Magnetic Resonance (NMR) are now the focus of intense research in the study of global embryo metabolism, aimed at determining their value as predictors of embryo viability, pregnancy rates [20–23], and even their potential ability to detect aneuploid embryos [24]. However, prospective randomized trials have previously found no benefit to using these methods to predict pregnancy rates [25–28].

In our previous study [29] we used a ultra-performance liquid chromatography coupled to mass spectrometry (UPLC-MS) untargeted metabolomic analysis to determine the differential global metabolic status of embryos coming from obese and normoweight IVF patients using spent media culture. Indeed, using this kind of approach, we obtained clear differences in the metabolism between these embryos.

In the present study, we used an untargeted metabolomic profiling approach based on UPLC-MS to compare the metabolic profile of embryos developed at low or atmospheric oxygen concentrations, and to then identify any metabolic profile that may differentiate them. Therefore, we aimed to analyze the metabolic footprint left specifically by the human cleavage-stage embryos, cultured under these two conditions, which also gave rise to a viable fetus with a heartbeat.

## Materials and Methods

### Participants and study design

This study was approved by the Institutional Review Board on the use of human subjects in research at the *Instituto Valenciano de Infertilidad* (IVI, Valencia). Our clinical investigation has been conducted according to the principles expressed in the Declaration of Helsinki. Informed written consent has been obtained from the participants. This prospective study was carried out within the context of a randomized controlled study performed in patients undergoing egg donation cycles with intracytoplasmic sperm injection (ICSI) IVF, with day-3 single/double embryo transfers that occurred between 2011 and 2012.

The current study consisted on using the spent culture media of 39 transferred and implanted embryos; 23 embryos from 13 patients in the 6% oxygen concentration group, and 16 embryos from 9 patients in the 20% oxygen concentration group.

### Stimulation protocol for donors

The controlled ovarian stimulation (COH) protocol for donors consisted of administering a daily dose of a GnRH (gonadotropin-releasing hormone)-agonist (triptorelin, 0.1 mg; Decapeptyl®, Ipsen Pharma, Barcelona, Spain) in the luteal phase after menses. COH was started with 225 IU of recombinant follicle stimulating hormone (FSH)/day (follitropin alfa; Gonal F®, Merck-Serono, Barclona Spain, or follitropin beta [rch]; Puregon®, Shering Plough, Madrid, Spain) or human menopausal gonadotropin (hMG; menotrophin; Menopur®, Ferring Pharmaceutical, Madrid, Spain). The daily dose was adjusted to ovarian response. Stimulation was prolonged until the mean diameter of the leading follicles was more than 18 mm. Recombinant human chorionic gonadotropin (rhCG; choriogonadotropin alfa; Ovitrelle®, Merk Serono) was administered and oocyte retrieval was carried out 36 h later. Anonymous donors were matched to egg recipients according to phenotype and blood groups. Both fresh oocytes and vitrified oocytes were used for egg donation purposes.

### Oocyte and embryo culture conditions

Four hours after egg retrieval, or two hours after oocyte warming [30, 31], oocytes were injected as previously described elsewhere [32]. ICSI was performed at 400 x magnification with a 1X7 Olympus microscope. Both injected oocytes and embryos were individually cultured in 50 μL drop, under oil of Sydney IVF cleavage media (COOK), which had been previously equilibrated overnight in the respective gas mixtures and then incubated in Sanyo MCI 5M incubators, set at either 6% O2 or 20% O2. In order to minimize the number of door openings, no more than two patients co-existed per incubator. An external quality control was performed daily with verified external probes for CO2, O2 and temperature measurements following the ISO 9000 standardized directives.

### Embryo morphology assessment

Nineteen hours post-insemination, oocytes were checked for fertilization. Embryo morphology was assessed on days 2 and 3 by considering the number of blastomeres, symmetry and granularity of blastomeres, type and percentage of fragmentation, presence of multinucleated blastomeres and degree of compaction. In accordance with these phenotype features, cleavage state embryos were scored according to four categories, A, B, C and D, which were partially described during the Istanbul consensus workshop on embryo assessment [33]. Of these four categories, A contained the top quality embryos and D was the lowest embryo quality category, implying less ability to implant.

### Sample collection

Each sample (40 μL) was obtained from the spent culture media of embryos developed either in the 6% oxygen group or in the 20% oxygen group. A total of 23 samples were collected from the 6% oxygen group, 16 from the 20% oxygen group, and 39 blank samples were used as a matched control media group. These samples were unused droplets made from the media from the same batch of embryos, cultured in the same conditions, but without the presence of an embryo.

Empty culture dishes where embryos had been growing for 48 ± 1 hours (from day 1 to day 3 of embryo development), were kept in the incubator for a maximum of 30 min after the embryos were removed for transfer. Sample collection was carried out by pipetting 40 μl out of the 50 μl drop of culture media Sydney IVF cleavage media (COOK into Eppendorf tubes (Eppendorf®, Oldenburg, Germany). Tubes containing culture media were spun at 10,000 rpm for 2 min and the supernatant was collected in a new Eppendorf tube to avoid debris or cell contamination and kept at -80°C until analyzed. The same procedure was used to collect matched media samples, consisting of 40 μL micro-drops of media cultured in the same conditions but without embryos.

### Metabolic profiling

Metabolic profiles were analyzed, as previously described, using methanol extraction followed by a UPLC-MS analysis (19). Briefly, samples were defrosted, mixed, and centrifuged; the sample was then diluted with 200 μL of methanol and mixed and stored at -20°C overnight. Following these samples were centrifuged at 13,000 rpm for 15 min; 200 μL of the supernatant was dried under vacuum, reconstituted in 100 μL of methanol, and transferred to vial for UPLC‐MS analysis. In order to avoid systematic bias in the analysis, all samples were randomized prior to the metabolite extraction procedure. Additionally, a pooled sample was also prepared and analyzed uniformly interspersed throughout the entire batch run (ten injections).

### UPLC‐MS analysis

The sample extracts were analyzed using a Waters Acquity UPLC coupled to a Waters QTof mass spectrometer (Waters Corp., Milford, USA). Chromatography was performed using HSS T3 C18 chemistry (1.0 × 100 mm) and a 1.8 μm particle-size column (Waters Corp.). The column was maintained at 40°C and eluted with a 13-min linear gradient. The mobile phase, at a flow rate of 140 μL/min, consisted of 100% solvent A (0.05% formic acid) for 1 min followed by an incremental increase of solvent B (acetonitrile containing 0.05% formic acid), which was taken up to 100% over the next 6 min, before returning to the initial composition mixture in readiness for the subsequent sample injection, which preceded a 45 s system recycle time. The volume of sample injected onto the column was 2 μL.

The mass spectrometer was operated with an electrospray (ESI) source held at 120°C. The nebulization gas was set to 600 L/h at a temperature of 350°C. The cone gas was set to 30 L/h with the capillary and cone voltages set to 3200 V and 30 V, and 2800 V and 50 V, in positive and negative modes respectively. The data acquisition rate was set to 0.5 s, with a 0.02 s inter‐scan delay. The mass range, 50‐1000 m/z was calibrated with cluster ions of sodium formate, using leucine enkephalin as an internal reference compound for instrumental drift correction (infused at 50 μL/min through an independent reference electrospray). An appropriate test mixture of standard compounds (Acetaminophen, Erythromicyns, Leucine-Enkephaline, Reserpine, Sulfaguanidine, Sulfadimethoxine, Terfenadine, and Val-Tyr-Val; all 5nM in water) was analyzed before and after the entire set of randomized, duplicated sample injections in order to examine the retention time stability, mass error, and sensitivity of the UPLC-MS system throughout the course of the run.

### Data Processing

All data were processed using the MarkerLynx application manager for MassLynx 4.1 software (Waters Corp.). The UPLC-MS data were peak‐detected and noise‐reduced in both the LC and MS domains such that only true analytical peaks were further processed by the software (e.g. noise spikes were rejected; signal detected must meet the criteria to be above the fixed intensity threshold level of 100 counts to be considered as a chromatographic peak). A list of intensities (chromatographic peak areas) of the peaks detected was then generated for the first sample, using the retention time (Rt) and mass-to-charge ratio (m/z) data pairs as the identifier for each peak. This process was repeated for each UPLC‐MS run. Once completed this process, the ion intensities for each peak detected within each sample were normalized to the sum of the peak intensities in that sample. Then, the overall quality of the analysis procedure was monitored using a pooled sample. All ion features included in the profiling analysis showed a percentage relative standard deviation (%RSD) lower than 30 in the ten replicated injections of the pooled sample.

In order to consider potential differences between culture media batches, a further normalization procedure was applied considering the corresponding matched blank samples, collected at the same conditions than the samples but without embryo. Therefore, the Rt‐m/z pairs detected in embryo-containing culture media were normalized to their levels in the corresponding blank culture medium samples. The resulting peak intensities formed a single matrix with Rt-m/z pairs for each sample normalized to their respective blank culture medium, which was considered for the following multivariate and univariate data analysis.

### Statistical analysis

#### Multivariate data analysis

**Fig 1:**
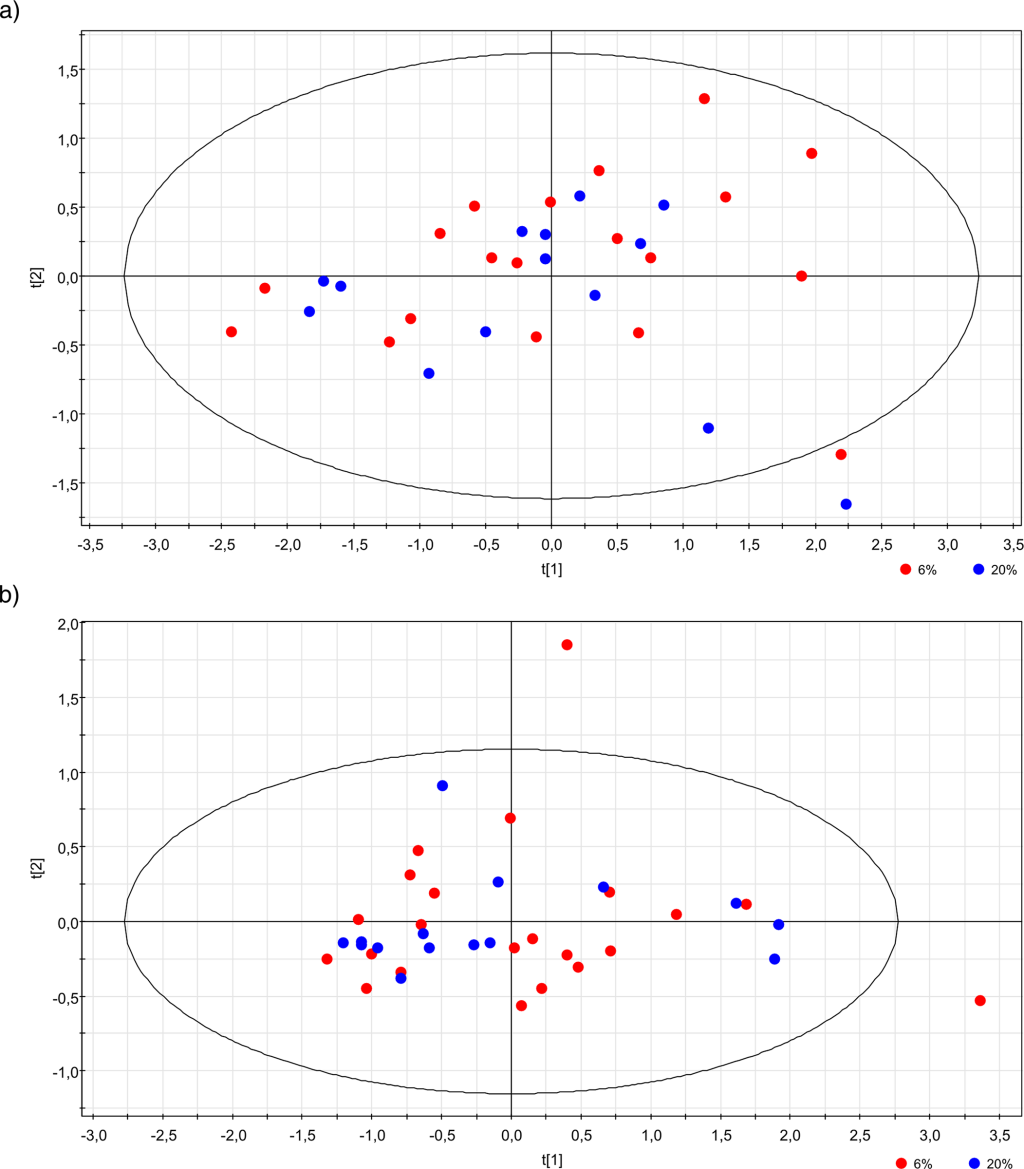
Principal components analysis (PCA) of untargeted global metabolomic profiles of the spent culture media from embryos cultured under 6% oxygen (red) and 20% oxygen (blue) concentrations. The first two principal components, t[1] and t[2], which represent the most important metabolic variation in the samples, are shown and were captured by Positive (R^2^X = 0.662 and Q^2^ = 0.519) (A) and Negative (R^2^X = 0.838 and Q^2^ = 0.492) analysis modes (B).

The first objective in the multivariate data analysis process was to reduce the dimensionality of the complex data set to enable easy visualization of any potential clustering of the groups of samples. This can be achieved using principal components analysis (PCA) [34] in which the data matrix is reduced to a series of principal components (PCs), i.e. each linear combination of the original Rt‐m/z pair peak areas. Each consecutive PC explains the maximum amount of variance possible, not explained by the previous PCs. Hence, the score plots of PCA analysis shown in Fig 1 (in which the first two principal components, t[1] and t[2], are displayed) show the most important metabolic variation in the samples captured by this analysis. The performance levels of the PCA models were evaluated using the R2X (variance modeled) and Q2 (cross‐validated R2X) parameters.

Supervised multivariate models were generated using the orthogonal partial least‐squares to latent structures (OPLS) method [35, 36]. An extra variable Y is created for each sample, which takes on the discrete values of 0 for 6% O_2_ concentration samples of 1 for 20% O_2_ concentration samples. Regression of this data allows new principal components to be calculated, which can then successively explain the maximum correlated X‐Y variance. Rotation of this OPLS model allows inter‐class (5%-20%) correlation to be captured in a single predictive component. The performance/validity of the OPLS models were evaluated using the R2Y (explained Y variance) and Q2 (cross validated R2Y) parameters.

#### Univariate data analysis

Univariate statistical analyses were also performed on the normalized dataset, using t‐tests for unpaired variables between the groups of samples, and applying Bonferroni correction for multiple testing.

## Results

### Demographics and embryo morphology

Regarding the egg donors, neither the age (26.4 ± 3.7 vs 26.0 ± 3.6) nor the BMI (24.5 ± 2.9 vs 23.0 ± 3.0) were different between the group of oocytes that were allocated to low or atmospheric oxygen concentration respectively.

The demographic features of the egg recipients and the embryo quality are shown in Table 1. No differences were observed regarding the etiology of the women included in the study.

**Table 1:** Demographics of egg recipients whose embryos were included in the study under both types of embryo culture conditions.

	6% CO2	20% CO2
Number of patients	13	9
Age of recipients (y)(mean ± SD)	40.6 ± 4.2	40.7 ± 4.3
BMI (mean ± SD)	20.8 ± 8,5	22,6 ± 7,6
Cause of female infertility		
Other	63.4%	61.6%
Age	17.8%	19.0%
Low response	5.0%	4.6%
Endometriosis	13.8%	14.8%.
% of cycles with fresh oocytes	61.5%	66.6%
Number of patients	13	9

Concerning morphological parameters of embryo quality, the average blastomere number, the percentage of fragmentation, and symmetry was comparable between both groups of embryos (Table 2).

**Table 2:** Embryology parameters of the analyzed embryos included in the study under both types of embryo culture conditions.

	6% CO2	20% CO2
Mumber of embryos	23	16
D3 cells (mean ± SD)	8.0 ± 0.8	8.3 ± 1.4
% Fragmentation(mean ± SD)	4.3 ± 3.7	2.6 ± 3.2
Symmetry	1.3 ± 0.6	1.6 ± 0.4

Mean number of blastomeres, percentage of fragmentation and symmetry were not statistically different.

### Global metabolite profiling

PCA analysis was performed in order to highlight the differences in the global metabolomic profiles between both oxygen concentrations groups. This type of analysis clusters the samples and represents them on a two dimensional space based on the differential relative concentration levels/amounts of all the measured metabolites. We observed no differences between the two groups (Fig 1), in both the positive and negative ion modes. In fact, both the 6% and 20% O_2_ populations failed to separate in this PCA analysis. Although supervised OPLS analysis was also performed, none of the OPLS models comparing 6% O_2_ vs. 20% O_2_ using positive and negative ion modes showed a positive predictability (data not shown). Based on these results, our multivariate statistical analyses of the untargeted metabolomic profiles of spent media coming from embryos cultured under either oxygen condition revealed that there were no significant differences in the metabolic profiles obtained from these day-3 embryos.

**Fig 2:**
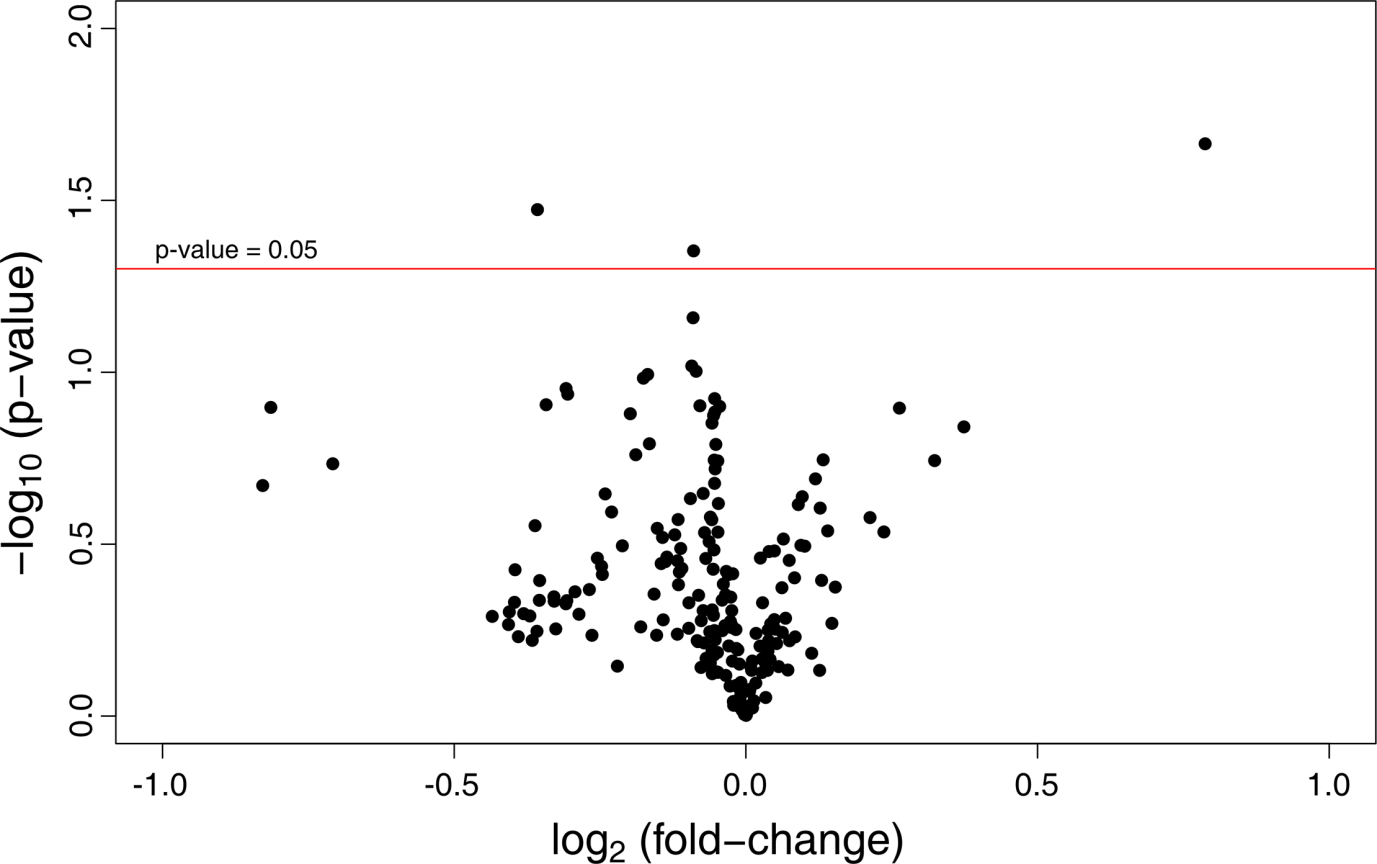
Volcano plots representing the relationship between fold change and statistical significance for all the spent culture media metabolites metabolites analyzed. The log2 average fold-change between the two groups is represented on the x-axis: 6% oxygen vs. 20% oxygen. The y-axis represents the -log10 *p*-value.

**Fig 3:**
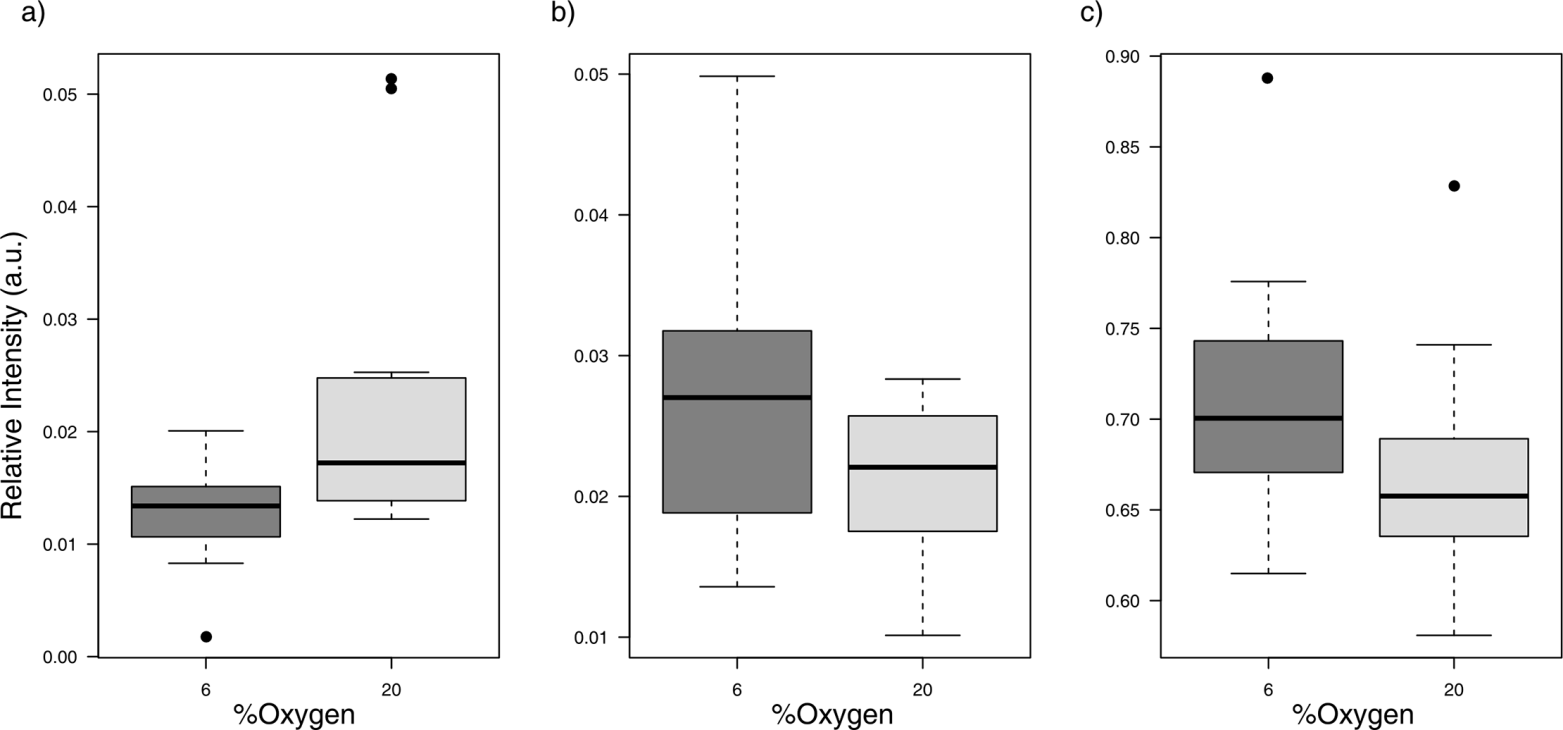
Box plots for the three metabolic features which were found to be significantly different after univariate analysis. Each metabolic feature is labeled based on their retention time and mass-to-charge ratio (termed Rt-m/z), 0.6267_671.7923 (A) 0.4981_151.0359 (B) and 0.583_92.9273 (C).

Finally, to make sure that no robust individual differences were obtained between the groups, we performed a univariate statistical analysis on all the ion features measured in our samples (S1 Fig). We tested the 103 metabolic features found in the spent media of our embryos and we found only three significant features that differed between the 6% and 20% oxygen concentration, representing approximately 3% of the total analyzed (Fig 2). However, the main drawback of this untargeted approach is that all the metabolic features were labeled based on their retention time and mass to charge ratio (termed Rt-m/z), and the identification of these Rt-m/z features is usually very time consuming and is not always possible. Nonetheless, the small group of three metabolic features (Rt-m/z pairs: 0.6267_671.7923 and 0.4981_151.0359 in the positive ion mode, and 0.583_92.9273 in the negative one) appeared to be significantly different, and showed a fold change (20% O_2_ vs. 6% O_2_) of 1.73, 0.78, and 0.93 respectively. The first one was higher in the 20% O_2_ embryos (1.7 [CI95%]; *p* = 0.02) and the other two were higher in the 6% O_2_ embryos (1.28 [CI95%]; *p* = 0.03, and 1.08 [CI95%]; *p* = 0.04 respectively). Box plots for these metabolites are shown in Fig 3.

**Fig 4:**
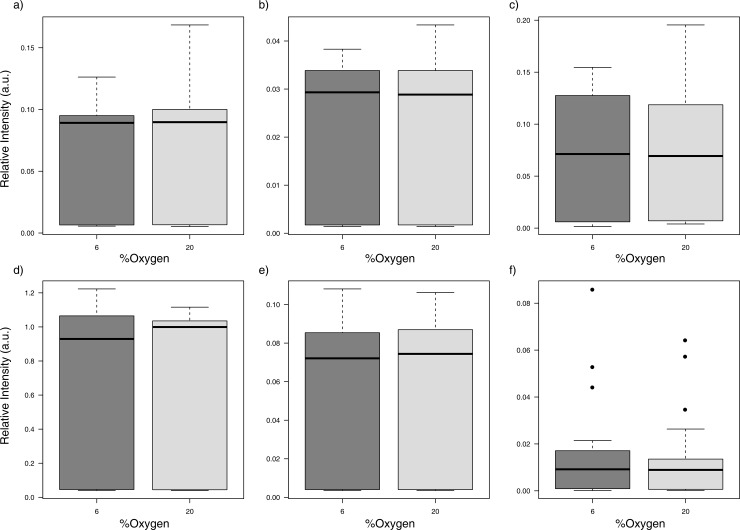
Box plots for selected metabolic features related to lipid, amino acids and carbohydrate metabolism. No significant differences were observed after univariate analysis. Araquidonic acid (20:4n-6) (A); alpha-linolenic acid (18:3n-6) (B); lysophosphatidylcholine LPC(18:1) (C); tryptophan (D); phenylalanine (E) and glucose and related hexoses metabolites (F).

We also checked other important metabolic features related to carbohydrate metabolism (glucose), amino acids, non-esterified fatty acids such as estearic, palmitic and oleic acids and glycerophospholipids, but all of them were not significantly altered depending on O_2_ concentration (Fig 4).

## Discussion

Under natural circumstances, the oxygen concentration surrounding both oocytes and human embryos ranges between 2% and 8% [37, 38]. In fact, during development embryos encounter two types of oxygen environments, the first one ranging from 5% to 8.6% O_2_ within the follicle and fallopian tubes [3], and the second 1.5–2% O_2_ inside the uterine cavity [1]. To mimic this situation in the IVF laboratory, two incubators set up to two different oxygen conditions depending on the developmental stage of the embryos should be used. However, the most common approach when culturing in vitro embryos is to use a fixed low oxygen concentration ranging from 5 to 6% throughout the whole process. Under such circumstances the actual oxygen concentration found in the culture media after culturing a 50 μL drop at 20% O_2_ is around 0.2 mM [39], whereas at the 6% concentration oxygen levels are around 60 μM following equilibration. Both oxygen concentrations are far above the actual oxygen needs of cleavage-stage human embryos which require approximately 5 Fmol/s [40].

Despite of the existence of evidences showing that the environmental stress created by using a higher oxygen tension during embryo culture may alter the metabolic activity of pre-implantation embryos [4, 41], the multivariate statistics we used in our analysis showed that cleavage-stage human embryos grown under both types of oxygen concentration culture conditions left a similar metabolic fingerprint. We failed to observe any change in the net depletion or release of relevant analytes, such as glucose, certain amino acids, fatty acids and glycerophospholipids by human cleavage-stage embryos under either type of culture conditions.

Pyruvate is the favorite substrate for cleavage stage mammalian embryos include the human and is able to support the development of human blastocysts [42–44], pyruvate and glucose uptake has previously been correlated with embryo viability [4, 45, 46] used more and, at least in mice, this factor is dependent on the oxygen concentration during in vitro culture [4]. The absence of differences in the glucose may have resulted from the fact that glucose uptake by cleavage stage human embryos unlike other carbohydrates such as the pyruvate, is actually smaller and may not change with the oxygen environment [47].

Concerning the amino acid uptake, this type of substrates are also oxidized by cleavage stage embryos to produce ATP [48]. It has been shown that embryos that develop from the early cleavage stage to form a blastocyst or which are chromosomally normal show a different profile of amino acid turnover from those that eventually arrest or have an abnormal chromosomal constitution [49–51]. Also In the mouse model, it has been demonstrated that a high oxygen concentration changes its uptake by increasing the amino acid utilization rate [4] and down regulates the production of proteins with a mass between 4 and 20 kDa [10].

Certain amino acids such as arginine, whose higher rates of depletion have been associated with poor embryo development [50], tyrosine, glycine, valine, and lysine turnover, associated with chromosomally abnormal embryos [49], or other polar amino acids related with live birth such as arsenine, glycine and leucine [52] were probably not detected in our assay. However, other amino acids such as tryptophane and phenylalanine that consistently appears in the culture during porcine preimplantation embryo development [53, 54] were similar on the two oxygen culture conditions.

Another group of metabolites analyzed in the present study were fatty acids and glycerophospholipids. Fatty acids are actually present in the human oocyte. They have been proposed as potential metabolites for ooytes and embryos, [55]; 80% of the fatty acids present in unfertilized human oocytes are saturated ones, of these, both the stearic and palmitic are the more predominant ones [56]. In our study, oxygen concentration during culture did not affect the final fatty acid concentration in spent conditioned culture [57]media. The reason for this may be because human embryos do not need to take any fatty acid from the media, first, because at the stage of development they do not have such high energy demands, as it has been calculated that fatty acid oxidation yields three times more energy that one molecule of glucose [58], and second because its uptake may not depend on external oxygen concentration.

Interestingly blank controls were positive for several fatty acids that were not described to be present in the Sydney IVF cleavage media. We hypothesize that these lipids could be carried by the human albumin present in the media, and could be eventually utilized by human embryos [55]. The addition of polyunsaturated and monounsaturated fatty acids in the culture media has shown to be involved in oocyte maturation as well as embryo development of different mammalian species, for review see [59].

We speculate that this metabolism modification in humans does not rely only on oxygen availability, at least at the early stages of development, and perhaps they may respond to oxygen variation only when embryo genome activation (EGA) starts to be fully activated, as have already been shown in the porcine [7]. As a matter of fact and based on the quire hypotesis, viable early mammalian embryos should be metabolically moderate rather that active [57], this lack of activity may explain our results.

Unfortunately, given the limitations of our methodological approach, this hypothesis cannot be fully demonstrated as the chromatographic method employed was not optimized for the analysis of polar metabolites such as pyruvate, lactate, and most of the amino acids. In metabolic profiling, there is no single platform or method to analyze the entire metabolome of a biological sample mainly due to the wide concentration range of metabolites coupled to their extensive chemical diversity. The current analytical methodology was optimized to provide maximum coverage over classes of compounds involved in key metabolic pathways, such as major phospholipids, non-esterified fatty acids, and some amino acids, while offering relatively high-throughput with minimal injection-to-injection carryover effects.

After univariate statistical analysis, only three non-identified analytes showed significant differences in their concentration levels between both groups. Moreover, due to the specificity of the statistical analysis in which we assume a 5% false positive rate, the sample dilution used (50 microL, and the limited time of the embryo culture (48h), we cannot be sure if these three ion features are indeed affected by the different O_2_ concentrations. It may be that they appeared to be significant due to the model we used, in which more than 100 analytes were analyzed, as none of the Rt-m/z pairs detected were found to be significantly altered after taking into account Bonferroni correction for multiple testing.

As a summary, despite no clear changes were observed on the global metabolic profile, other specific molecules could have escaped from our deep analysis. Furthermore, we must not forget other possible reasons for caution when interpreting our results. As only embryos that yielded viable pregnancies at least up to the 12 week of gestation were analyzed, we cannot rule out the possibility that the similarities between the embryos from both culture conditions were due to the fact that only embryos with at least comparable metabolism successfully implanted. Lastly, larger number of subjects will be needed to substantiate these preliminary results.

## Supporting Information

S1 Fig Raw data of the metabolic ion features (Rt‐m/z pairs) in the spent media of embryos cultured in low and high oxygen concentration, normalised to the sum of the peak intensities in each sample and to their levels in the corresponding blank culture medium samples. Data provided for detected ion features in positive and negative ion modes.
(XLSX)

## References

[1] Fischer B, Bavister BD. Oxygen tension in the oviduct and uterus of rhesus monkeys, hamsters and rabbits. Journal of reproduction and fertility. 1993;99(2):673–9. 8107053 10.1530/jrf.0.0990673

[2] Fischer B, Kunzel W, Kleinstein J, Gips H. Oxygen tension in follicular fluid falls with follicle maturation. European journal of obstetrics, gynecology, and reproductive biology. 1992;43(1):39–43. 1737607 10.1016/0028-2243(92)90241-p

[3] Van Blerkom J, Antczak M, Schrader R. The developmental potential of the human oocyte is related to the dissolved oxygen content of follicular fluid: association with vascular endothelial growth factor levels and perifollicular blood flow characteristics. Hum Reprod. 1997;12(5):1047–55. 9194664 10.1093/humrep/12.5.1047

[4] Wale PL, Gardner DK. Oxygen regulates amino acid turnover and carbohydrate uptake during the preimplantation period of mouse embryo development. Biology of reproduction. 2012;87(1):24, 1–8. 10.1095/biolreprod.112.100552 22553221

[5] Bontekoe S, Mantikou E, van Wely M, Seshadri S, Repping S, Mastenbroek S. Low oxygen concentrations for embryo culture in assisted reproductive technologies. Cochrane Database Syst Rev. 2012;7:CD008950 10.1002/14651858.CD008950.pub2 22786519 PMC11683526

[6] Bauer BK, Isom SC, Spate LD, Whitworth KM, Spollen WG, Blake SM, et al Transcriptional profiling by deep sequencing identifies differences in mRNA transcript abundance in in vivo-derived versus in vitro-cultured porcine blastocyst stage embryos. Biology of reproduction. 2010;83(5):791–8. 10.1095/biolreprod.110.085936 20668257

[7] Redel BK, Brown AN, Spate LD, Whitworth KM, Green JA, Prather RS. Glycolysis in preimplantation development is partially controlled by the Warburg Effect. Molecular reproduction and development. 2012;79(4):262–71. 10.1002/mrd.22017 22213464

[8] Whitworth KM, Agca C, Kim JG, Patel RV, Springer GK, Bivens NJ, et al Transcriptional profiling of pig embryogenesis by using a 15-K member unigene set specific for pig reproductive tissues and embryos. Biology of reproduction. 2005;72(6):1437–51. 10.1095/biolreprod.104.037952 15703372

[9] Gardner DK, Hamilton R, McCallie B, Schoolcraft WB, Katz-Jaffe MG. Human and mouse embryonic development, metabolism and gene expression are altered by an ammonium gradient in vitro. Reproduction. 2013;146(1):49–61. 10.1530/REP-12-0348 23613618

[10] Katz-Jaffe MG, Linck DW, Schoolcraft WB, Gardner DK. A proteomic analysis of mammalian preimplantation embryonic development. Reproduction. 2005;130(6):899–905. 10.1530/rep.1.00854 16322549

[11] Edwards JL, Ealy AD, Hansen PJ. Regulation of heat shock protein 70 synthesis by heat shock in the preimplantation murine embryo. Theriogenology. 1995;44(3):329–37. 16727733 10.1016/0093-691x(95)00188-e

[12] Wrenzycki C, Herrmann D, Keskintepe L, Martins A Jr., Sirisathien S, Brackett B, et al Effects of culture system and protein supplementation on mRNA expression in pre-implantation bovine embryos. Hum Reprod. 2001;16(5):893–901. 11331635 10.1093/humrep/16.5.893

[13] Correa GA, Rumpf R, Mundim TC, Franco MM, Dode MA. Oxygen tension during in vitro culture of bovine embryos: effect in production and expression of genes related to oxidative stress. Animal reproduction science. 2008;104(2–4):132–42. 10.1016/j.anireprosci.2007.02.002 17350772

[14] Pucci O, Qualls C, Battisti-Charbonney A, Balaban DY, Fisher JA, Duffin J, et al Human skin hypoxia modulates cerebrovascular and autonomic functions. PloS one. 2012;7(10):e47116 10.1371/journal.pone.0047116 23056597 PMC3466185

[15] Robinson CM, Neary R, Levendale A, Watson CJ, Baugh JA. Hypoxia-induced DNA hypermethylation in human pulmonary fibroblasts is associated with Thy-1 promoter methylation and the development of a pro-fibrotic phenotype. Respiratory research. 2012;13:74 10.1186/1465-9921-13-74 22938014 PMC3519562

[16] Shahrzad S, Bertrand K, Minhas K, Coomber BL. Induction of DNA hypomethylation by tumor hypoxia. Epigenetics: official journal of the DNA Methylation Society. 2007;2(2):119–25. 10.4161/epi.2.2.4613 17965619

[17] Singh R, Sinclair KD. Metabolomics: approaches to assessing oocyte and embryo quality. Theriogenology. 2007;68 Suppl 1:S56–62. 10.1016/j.theriogenology.2007.04.007 17490741

[18] Nicholson JK, Lindon JC, Holmes E. 'Metabonomics': understanding the metabolic responses of living systems to pathophysiological stimuli via multivariate statistical analysis of biological NMR spectroscopic data. Xenobiotica; the fate of foreign compounds in biological systems. 1999;29(11):1181–9. 10.1080/004982599238047 10598751

[19] Dominguez F, Castello D, Remohi J, Simon C, Cobo A. Effect of vitrification on human oocytes: a metabolic profiling study. Fertility and sterility. 2013;99(2):565–72. 10.1016/j.fertnstert.2012.09.034 23102858

[20] Brison DR, Hollywood K, Arnesen R, Goodacre R. Predicting human embryo viability: the road to non-invasive analysis of the secretome using metabolic footprinting. Reproductive biomedicine online. 2007;15(3):296–302. 17854527 10.1016/s1472-6483(10)60342-2

[21] Seli E, Bruce C, Botros L, Henson M, Roos P, Judge K, et al Receiver operating characteristic (ROC) analysis of day 5 morphology grading and metabolomic Viability Score on predicting implantation outcome. Journal of assisted reproduction and genetics. 2011;28(2):137–44. 10.1007/s10815-010-9501-9 21063765 PMC3059520

[22] Seli E, Sakkas D, Scott R, Kwok SC, Rosendahl SM, Burns DH. Noninvasive metabolomic profiling of embryo culture media using Raman and near-infrared spectroscopy correlates with reproductive potential of embryos in women undergoing in vitro fertilization. Fertility and sterility. 2007;88(5):1350–7. 10.1016/j.fertnstert.2007.07.1390 17923129

[23] Nadal-Desbarats L, Veau S, Blasco H, Emond P, Royere D, Andres CR, et al Is NMR metabolic profiling of spent embryo culture media useful to assist in vitro human embryo selection? MAGMA. 2013;26(2):193–202. 10.1007/s10334-012-0331-x 22878530

[24] Sanchez-Ribas I, Riqueros M, Vime P, Puchades-Carrasco L, Jonsson T, Pineda-Lucena A, et al Differential metabolic profiling of non-pure trisomy 21 human preimplantation embryos. Fertility and sterility. 2012;98(5):1157–64 e1-2. 10.1016/j.fertnstert.2012.07.1145 22959456

[25] Hardarson T, Ahlstrom A, Rogberg L, Botros L, Hillensjo T, Westlander G, et al Non-invasive metabolomic profiling of Day 2 and 5 embryo culture medium: a prospective randomized trial. Hum Reprod. 2012;27(1):89–96. 10.1093/humrep/der373 22068638

[26] Kirkegaard K, Svane AS, Nielsen JS, Hindkjaer JJ, Nielsen NC, Ingerslev HJ. Nuclear magnetic resonance metabolomic profiling of Day 3 and 5 embryo culture medium does not predict pregnancy outcome in good prognosis patients: a prospective cohort study on single transferred embryos. Hum Reprod. 2014;29(11):2413–20. 10.1093/humrep/deu236 25256566

[27] Rust M. Scientists ponder ethics of genetic era. American medical news. 1984;27(15):3, 12. 11653548

[28] Vergouw CG, Heymans MW, Hardarson T, Sfontouris IA, Economou KA, Ahlstrom A, et al No evidence that embryo selection by near-infrared spectroscopy in addition to morphology is able to improve live birth rates: results from an individual patient data meta-analysis. Hum Reprod. 2014;29(3):455–61. 10.1093/humrep/det456 24408316

[29] Bellver J, De Los Santos MJ, Alama P, Castello D, Privitera L, Galliano D, et al Day-3 embryo metabolomics in the spent culture media is altered in obese women undergoing in vitro fertilization. Fertility and sterility. 2015;103(6):1407–15 e1. 10.1016/j.fertnstert.2015.03.015 25935493

[30] Cobo A, Meseguer M, Remohi J, Pellicer A. Use of cryo-banked oocytes in an ovum donation programme: a prospective, randomized, controlled, clinical trial. Hum Reprod. 2010;25(9):2239–46. 10.1093/humrep/deq146 20591872

[31] Cobo A, Remohi J, Chang CC, Nagy ZP. Oocyte cryopreservation for donor egg banking. Reproductive biomedicine online. 2011;23(3):341–6. Epub 2011/07/20. 10.1016/j.rbmo.2011.05.014 21767989

[32] Rubio C, Minguez Y, De Los Santos MJ, Ruiz A, Romero J. Intracytoplasmic sperm injection (ICSI). Frontiers in bioscience: a journal and virtual library. 1997;2:f1. 9159252 10.2741/a234

[33] Bauereis B, Haskins WE, Lebaron RG, Renthal R. Proteomic insights into the protective mechanisms of an in vitro oxidative stress model of early stage Parkinson's disease. Neuroscience letters. 2011;488(1):11–6. Epub 2010/11/09. 10.1016/j.neulet.2010.10.071 21056633 PMC3010496

[34] Joliffe IT. In: Springer, editor. Principal component Analysis 2 ed: New York: Springer; 2002.

[35] Bylesjo M, Rantalainen M, Nicholson JK, Holmes E, Trygg J. K-OPLS package: kernel-based orthogonal projections to latent structures for prediction and interpretation in feature space. BMC bioinformatics. 2008;9:106 10.1186/1471-2105-9-106 18284666 PMC2323673

[36] Wiklund S, Johansson E, Sjostrom L, Mellerowicz EJ, Edlund U, Shockcor JP, et al Visualization of GC/TOF-MS-based metabolomics data for identification of biochemically interesting compounds using OPLS class models. Analytical chemistry. 2008;80(1):115–22. 10.1021/ac0713510 18027910

[37] Ottosen LD, Hindkaer J, Husth M, Petersen DE, Kirk J, Ingerslev HJ. Observations on intrauterine oxygen tension measured by fibre-optic microsensors. Reproductive biomedicine online. 2006;13(3):380–5. 16984770 10.1016/s1472-6483(10)61443-5

[38] Yedwab GA, Paz G, Homonnai TZ, David MP, Kraicer PF. The temperature, pH, and partial pressure of oxygen in the cervix and uterus of women and uterus of rats during the cycle. Fertility and sterility. 1976;27(3):304–9. 3445 10.1016/s0015-0282(16)41722-x

[39] Trimarchi JR, Liu L, Porterfield DM, Smith PJ, Keefe DL. Oxidative phosphorylation-dependent and -independent oxygen consumption by individual preimplantation mouse embryos. Biology of reproduction. 2000;62(6):1866–74. 10819794 10.1095/biolreprod62.6.1866

[40] Tejera A, Herrero J, Viloria T, Romero JL, Gamiz P, Meseguer M. Time-dependent O2 consumption patterns determined optimal time ranges for selecting viable human embryos. Fertility and sterility. 2012;98(4):849–57 e1-3. 10.1016/j.fertnstert.2012.06.040 22835446

[41] Kumar P, Verma A, Kumar M, De S, Kumar R, Datta TK. Expression pattern of glucose metabolism genes correlate with development rate of buffalo oocytes and embryos in vitro under low oxygen condition. Journal of assisted reproduction and genetics. 2015;32(3):471–8. Epub 2015/01/13. 10.1007/s10815-014-0418-6 25578537 PMC4363242

[42] Conaghan J, Handyside AH, Winston RM, Leese HJ. Effects of pyruvate and glucose on the development of human preimplantation embryos in vitro. Journal of reproduction and fertility. 1993;99(1):87–95. 8283458 10.1530/jrf.0.0990087

[43] Hardy K, Hooper MA, Handyside AH, Rutherford AJ, Winston RM, Leese HJ. Non-invasive measurement of glucose and pyruvate uptake by individual human oocytes and preimplantation embryos. Hum Reprod. 1989;4(2):188–91. 2918073 10.1093/oxfordjournals.humrep.a136869

[44] Leese HJ, Hooper MA, Edwards RG, Ashwood-Smith MJ. Uptake of pyruvate by early human embryos determined by a non-invasive technique. Hum Reprod. 1986;1(3):181–2. 3624425 10.1093/oxfordjournals.humrep.a136376

[45] Gardner DK, Leese HJ. Non-invasive measurement of nutrient uptake by single cultured pre-implantation mouse embryos. Hum Reprod. 1986;1(1):25–7. 3455417 10.1093/oxfordjournals.humrep.a136336

[46] Gardner DK, Leese HJ. The role of glucose and pyruvate transport in regulating nutrient utilization by preimplantation mouse embryos. Development. 1988;104(3):423–9. 3076862 10.1242/dev.104.3.423

[47] Conaghan J, Hardy K, Handyside AH, Winston RM, Leese HJ. Selection criteria for human embryo transfer: a comparison of pyruvate uptake and morphology. Journal of assisted reproduction and genetics. 1993;10(1):21–30. 8499675 10.1007/BF01204436

[48] Leese HJ, Baumann CG, Brison DR, McEvoy TG, Sturmey RG. Metabolism of the viable mammalian embryo: quietness revisited. Molecular human reproduction. 2008;14(12):667–72. 10.1093/molehr/gan065 19019836 PMC2639445

[49] Picton HM, Elder K, Houghton FD, Hawkhead JA, Rutherford AJ, Hogg JE, et al Association between amino acid turnover and chromosome aneuploidy during human preimplantation embryo development in vitro. Molecular human reproduction. 2010;16(8):557–69. 10.1093/molehr/gaq040 20571076 PMC2907220

[50] Houghton FD, Hawkhead JA, Humpherson PG, Hogg JE, Balen AH, Rutherford AJ, et al Non-invasive amino acid turnover predicts human embryo developmental capacity. Hum Reprod. 2002;17(4):999–1005. 11925397 10.1093/humrep/17.4.999

[51] Houghton FD, Leese HJ. Metabolism and developmental competence of the preimplantation embryo. European journal of obstetrics, gynecology, and reproductive biology. 2004;115 Suppl 1:S92–6. 10.1016/j.ejogrb.2004.01.019 15196724

[52] Brison DR, Houghton FD, Falconer D, Roberts SA, Hawkhead J, Humpherson PG, et al Identification of viable embryos in IVF by non-invasive measurement of amino acid turnover. Hum Reprod. 2004;19(10):2319–24. 10.1093/humrep/deh409 15298971

[53] Booth PJ, Watson TJ, Leese HJ. Prediction of porcine blastocyst formation using morphological, kinetic, and amino acid depletion and appearance criteria determined during the early cleavage of in vitro-produced embryos. Biology of reproduction. 2007;77(5):765–79. 10.1095/biolreprod.107.062802 17652665

[54] Booth PJ, Humpherson PG, Watson TJ, Leese HJ. Amino acid depletion and appearance during porcine preimplantation embryo development in vitro. Reproduction. 2005;130(5):655–68. 10.1530/rep.1.00727 16264095

[55] Sturmey RG, Reis A, Leese HJ, McEvoy TG. Role of fatty acids in energy provision during oocyte maturation and early embryo development. Reproduction in domestic animals = Zuchthygiene. 2009;44 Suppl 3:50–8. 10.1111/j.1439-0531.2009.01402.x 19660080

[56] Matorras R, Ruiz JI, Mendoza R, Ruiz N, Sanjurjo P, Rodriguez-Escudero FJ. Fatty acid composition of fertilization-failed human oocytes. Hum Reprod. 1998;13(8):2227–30. 9756301 10.1093/humrep/13.8.2227

[57] Baumann CG, Morris DG, Sreenan JM, Leese HJ. The quiet embryo hypothesis: molecular characteristics favoring viability. Molecular reproduction and development. 2007;74(10):1345–53. 10.1002/mrd.20604 17342740

[58] Dunning KR, Russell DL, Robker RL. Lipids and oocyte developmental competence: the role of fatty acids and beta-oxidation. Reproduction. 2014;148(1):R15–27. 10.1530/REP-13-0251 24760880

[59] McKeegan PJ, Sturmey RG. The role of fatty acids in oocyte and early embryo development. Reproduction, fertility, and development. 2011;24(1):59–67. 10.1071/RD11907 22394718

